# Body ownership and experiential ownership in the self-touching illusion

**DOI:** 10.3389/fpsyg.2014.01591

**Published:** 2015-01-20

**Authors:** Caleb Liang, Si-Yan Chang, Wen-Yeo Chen, Hsu-Chia Huang, Yen-Tung Lee

**Affiliations:** ^1^Department of Philosophy, National Taiwan UniversityTaipei, Taiwan; ^2^Graduate Institute of Brain and Mind Sciences, National Taiwan UniversityTaipei, Taiwan

**Keywords:** body ownership, experiential ownership, self-as-object, self-as-subject, self-touching illusion, pre-reflective immunity, bodily self-consciousness

## Abstract

We investigate two issues about the subjective experience of one's body: first, is the experience of owning a full-body fundamentally different from the experience of owning a body-part?Second, when I experience a bodily sensation, does it guarantee that I cannot be wrong about whether it is me who feels it? To address these issues, we conducted a series of experiments that combined the rubber hand illusion (RHI) and the “body swap illusion.” The subject wore a head mounted display (HMD) connected with a stereo camera set on the experimenter's head. Sitting face to face, they used their right hand holding a paintbrush to brush each other's left hand. Through the HMD, the subject adopted the experimenter's first-person perspective (1PP) as if it was his/her own 1PP: the subject watched either the experimenter's hand from the adopted 1PP, and/or the subject's own hand from the adopted third-person perspective (3PP) in the opposite direction (180°), or the subject's full body from the adopted 3PP (180°, with or without face). The synchronous full-body conditions generate a “self-touching illusion”: many participants felt that “I was brushing my own hand!” We found that (1) the sense of body-part ownership and the sense of full-body ownership are not fundamentally different from each other; and (2) our data present a strong case against the mainstream philosophical view called the immunity principle (IEM). We argue that it is possible for misrepresentation to occur in the subject's sense of “experiential ownership” (the sense that I am the one who is having this bodily experience). We discuss these findings and conclude that not only the sense of body ownership but also the sense of experiential ownership call for further interdisciplinary studies.

## Introduction

Many daily experiences involve the sense of *body ownership*, which concerns what it is like to feel this hand or body *as mine*. Walking into a coffee shop, I quickly get a cup of cappuccino and take a sip to enjoy the nice taste and aroma. I experience the hand holding the cup as *my* hand, and I experience this particular body that just walked in as *my* body. Although the sense of body ownership has been studied by many groups in recent years, two key issues are still to be addressed. First, what is the relationship between the sense of body-part ownership and the sense of full-body ownership? Is the latter fundamentally different from the former? Or is the difference only a matter of degree? The second issue concerns: *who* is undergoing the experiences that occur in this particular body or body-part? By walking in and taking the sip, I not only experience this hand and body as mine, but I also have an implicit sense that I am the unique subject of those experiences that involve my body or body parts. For instance, I have an implicit sense that it is *me* who is experiencing the specific aroma and taste of cappuccino, it is me who is having the tactile sensations of holding the coffee mug, and it is me who is experiencing this particular body that just walked into the coffee shop. We will call this the sense of *experiential ownership*. The issue that we intend to investigate is: can one's sense of experiential ownership go wrong?

The sense of body ownership and the sense of experiential ownership correspond to the philosophical distinction between the sense of *self-as-object* and the sense of *self-as-subject* (Wittgenstein, 1958; Shoemaker, 1968; Gallagher, 2012). Wittgenstein once made a famous distinction between using the first-person pronoun “I” *as-object* and using same term *as-subject*. He says: “It is possible that, say in an accident, I should feel a pain in my arm, see a broken arm at my side, and think it is mine, when really it is my neighbor's … On the other hand, there is no question of recognizing a person when I say I have toothache. To ask ‘are you sure it is *you* who have pains?’ would be nonsensical” (1958, p. 67). The idea is that when one is conscious of oneself-as-object, error is always possible. However, when one is conscious of oneself-as-subject, a specific type of mistake is impossible. Shoemaker (1968) has articulated this idea by explaining that we are “immune to error through misidentification relative to the first-person pronouns” (IEM). Focusing on the case of phenomenal state, IEM states that when I am aware of a phenomenal state through first-personal access, such as introspection, somatosensation, proprioception, etc., I *cannot be wrong* about whether it is I who feels it.

The characterization of self-as-object above fits well with our current knowledge about body ownership. Researchers have shown how misrepresentations may occur in one's sense of body ownership. This fits well with the view that “I”-as object and consciousness of self-as-object can be mistaken. In the RHI, watching a rubber hand being stroked synchronously with one's own unseen hand causes many subjects to feel as if the rubber hand is their own hand (Botvinick and Cohen, 1998; Tsakiris and Haggard, 2005). Researchers have studied not only body-part but also full-body illusions. In the full-body experiments by Lenggenhager et al. (2007), many subjects experienced the illusion that the virtual body was their own, and that they saw themselves from outside the body (cf. Ehrsson, 2007 for a different set-up). In the body swap illusion, many subjects felt that another person's whole body became their own and reported that “I was shaking hands with myself!” (Petkova and Ehrsson, 2008, p. 5). These experiments have been widely used as paradigms for studying the sense of body ownership (Bertamini et al., 2011; Rohde et al., 2011; Pfeiffer et al., 2013; Salomon et al., 2013; van Doorn et al., 2014).

In light of these studies, the first issue can be stated as follows: Is the sense of body ownership involved in full-body illusions fundamentally different from that involved in the body-part illusion? Or is the difference only a matter of degree? On the one hand, Blanke and Metzinger (2009) propose that the most basic sense of self, i.e., what they call *minimal phenomenal selfhood* (MPS), is “experienced as a single feature, namely a coherent representation of the whole, spatially situated body—and not as multiple representations of separate body parts” (p. 9). The key features of MPS, including self-identification, self-location and first-person perspective are not modulated during the RHI. So Blanke and Metzinger believe that MPS can be illuminated only by investigating the sense of full-body ownership. This suggests that the sense of full-body ownership is fundamentally distinct from the sense of body-part ownership. On the other hand, Tsakiris (2010) focuses primarily on the RHI and holds that “the necessary conditions for the experience of ownership over a body-part seem to be the same as the ones involved in the experience of ownership for full bodies” (p. 710). Also, Petkova et al. (2011b) in an fMRI study suggest that “the unitary experience of owning an entire body is produced by neuronal populations that integrate multisensory information across body segments” (p. 1118). These views lean toward the position that there is no essential difference between the sense of body-part ownership and the sense of full-body ownership. We think that, in order to solve this issue, the body-part and full-body illusions should not be treated separately. So in this study we combine both types of illusions. We test the hypothesis that the sense of body-part ownership and the sense of full-body ownership are not fundamentally distinct from each other. This psychophysical hypothesis, if correct, may provide a useful guide for investigating the relevant neural mechanisms.

In contrast to our current knowledge of body ownership, experiential ownership is almost neglected by researchers. To clarify the second issue, it will be very useful to distinguish between the *fact* and the *sense* of experiential ownership. Consider the simple example again. On the one hand, it is a fact that right now it is me, not you, who is the subject of this particular experience. Call this the *fact* of experiential ownership. This fact is objective because, for every conscious experience we can ask “who is the subject of that experience?” and there is a fact about it. On the other hand, this fact is connected with a first-personal perspective: in taking the sip, I have an implicit sense that it is me who is having this experience. Call this the *sense* of experiential ownership. It is subjective in that one can experience oneself as the subject simply by experiencing phenomenal states; the former is a part of the latter. We suggest that the sense of self-as-subject introduced above can be captured by the sense of experiential ownership.

Now the second issue is: can misrepresentation occur in one's sense of experiential ownership? Can one's sense of experiential ownership misrepresent the relevant fact of experiential ownership? Influenced by Wittgenstein and Shoemaker, most philosophers believe that this is a purely conceptual or semantic issue, and the answer is negative (Coliva, 2006; cf. also papers in Prosser and Récanati, 2012). We are skeptical about this mainstream position, and propose that the sense of experiential ownership is open to empirical investigations. Our hypothesis is that, the fact of experiential ownership can be misrepresented by the subject's sense of experiential ownership. *Pace* Wittgenstein, sometimes it makes perfect sense to ask “are you sure it is you who is experiencing so-and-so?” and that Shoemaker's immunity principle (IEM), or at least some versions of it, fails to hold.

To address these two issues and test our hypotheses, we conducted a series of experiments that combined the rubber hand illusion (RHI, Botvinick and Cohen, 1998; Tsakiris and Haggard, 2005) and the “body swap illusion” (Petkova and Ehrsson, 2008). By manipulating the participant's visual perspective and allowing the participant to interact with the experimenter, many subjects experienced what we call the “self-touching illusion”: the subject felt that “I was brushing my own hand!” The subject was touching someone and being touched at the same time, as well as watching his/her own body in front of him/herself. This subject-experimenter interaction makes the illusion quite different from both the standard RHI and full-body illusions (FBI, Ehrsson, 2007; Lenggenhager et al., 2007). As we will see below, the self-touching experiments enabled us to compare the sense of full-body ownership with the sense of body-part ownership. Moreover, they created a situation wherein, subjectively, it was not totally clear whether it was me or someone else who felt the touch.

## Methods

### Materials and participants

In this study, we used a head mounted display (HMD, Sony HMZ-T1) and a stereo camera (Sony HDR-TD20V). The skin conductance responses (SCR) were recorded with a Data Acquisition Unit-MP35 (Biopac Systems, Inc. USA). For questionnaires, we used a Likert scale from “strongly disagree” (−3) to “strongly agree” (+3). The questionnaire statements are randomly distributed and can be divided into the following categories: body-part ownership, full-body ownership, touch referral, agency, self-touching illusion, experiential ownership, and double body effect (Table 1). We conducted three experiments, each with four conditions. See Table 2 below for the details of the participants. All participants gave written consent prior to the experiments. This study was approved by the Research Ethics Committee of National Taiwan University (NTU-REC: 201310HS026).

**Table 1:** **The questionnaires consisting of 13 statements divided into seven categories**.

**Category**		
Body-part ownership	Q1.	It felt as if the hand seen on the screen was my hand
Touch fererral	Q2.	It seemed as if the touch I felt was on the hand brushed by the paintbrush on te screen
	Q11.	It seemed as if the touch I felt was on the body in front of me
Agency	Q3.	It felt as if I could control the hand holding the paintbrush on the screen
	Q8.	It felt as if I could control the body in front of me
Full-body ownership	Q6.	It felt as if the body in front of me was mine.
	Q7.	It felt as if I was sitting in front of me
Self-touching illusion	Q4.	It felt as if I was brushing my own hand
	Q5.	The person whom I brushed was me, not someone else
Experiential ownership	Q9.	It was me who felt being brushed, not someone else
	Q10.	The person who felt being brushed was not me
Double body effect	Q12.	It felt as if I had two bodies
	Q13.	It felt as if I was looking at myself from the opposite side

*Statements in questionnaires. The questionnaires were in Chinese when presented to participants. Here are the English translations, and the wordings of some statements were slightly adjusted to fit the different HMD images across experiments. The questionnaires for the full-body conditions did not contain body-part statements Q1–Q3*.

**Table 2:** **Overview of experiments**.

**Experiment**	**Description**		**Measures taken**	**Participants (n)**	**Statistics**
Experiment 1	Cond. 1: Sync.	Full body, face, active hand	Questionnaire (FB1)	38 ( 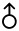 21)	*t*-test (Sync. Vs. Async)
				*M* = 21.55 ± 2.90	
	Cond. 2: Async.	Full body, face, active hand	Questionnaire	35 ( 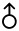 18)	
				*M* = 21.23 ± 1.66	
	Cond. 3: Sync.	Full body, face, active hand	SCR	15 ( 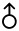 7)	*t*-test (Sync. Vs. Async)
				*M* = 22.13 ± 3.14	
	Cond. 4: Async.	Full body, face, active hand	SCR	15 ( 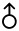 6)	
				*M* = 22.47 ± 3.02	
Experiment 2	Cond. 1: Sync.	Full body, no face, passive hand	Questionnaire (FB2)	28 ( 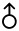 20)	*t*-test (Sync. Vs. Async)
				*M* = 23.75 ± 3.63	
	Cond. 2: Async.	Full body, no face, passive hand	Questionnaire	14 ( 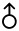 11)	
				*M* = 23.43 ± 3.80	
	Cond. 3: Sync.	Full body, no face, passive hand	SCR	13 ( 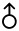 9)	*t*-test (Sync. Vs. Async)
				*M* = 24.08 ± 3.52	
	Cond. 4: Async.	Full body, no face, passive hand	SCR	13 ( 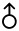 10)	
				*M* = 23.69 ± 3.82	
Experiment 3	Cond. 1: Sync.	Full body, no face, no hand	Questionnaire (FB3)	24 ( 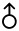 15)	ANOVA
				*M* = 21.50 ± 2.73	
	Cond. 2: Sync.	Full body, no face, active hand	Questionnaire (FB4)	27 ( 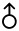 13)	ANOVA
				*M* = 21.52 ± 3.33	
	Cond. 3: Sync.	1PP passive hand	Questionnaire (BP1)	25 ( 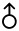 10)	ANOVA
				*M* = 21.48 ± 2.26	
	Cond. 4: Sync.	1PP passive hand & 3PP active hand	Questionnaire (BP2)	27 ( 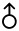 16)	ANOVA
				*M* = 21.44 ± 2.69	

### Procedure

**Figure 1:**
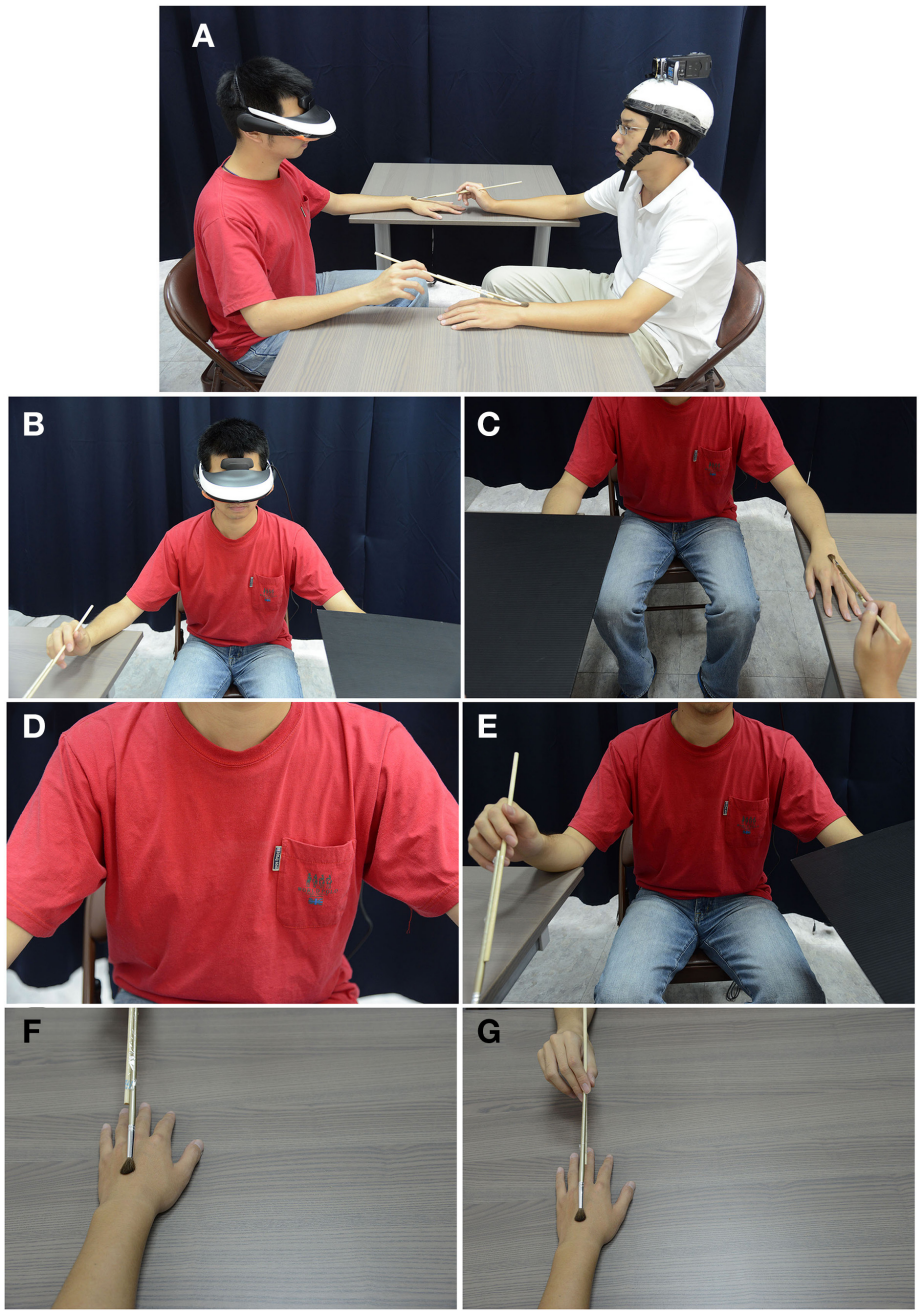
**Experimental set-up. (A)** The Basic Setting. The participant wore a HMD connected with a stereo camera positioned on the experimenter's head. They sat face to face to brush each other's left hand with a paintbrush held in their right hand. **(B)** illustrates what participants saw through the HMD in Experiment 1. The participants saw the front side of their virtual body from the adopted 3PP, including torso, legs, face, and their right hand holding a paintbrush. The synchronous condition measured by questionnaire will be called FB1. **(C)** illustrates what the participants saw via the HMD in Experiment 2. The participants saw their own virtual torso, legs, but not the face. They also saw their left hand being touched by a paintbrush held by the experimenter's hand, which was seen from the adopted 1PP. The synchronous condition measured by questionnaire will be called FB2. **(D)** FB3 in Experiment 3: through the HMD, the participants saw the front side of their virtual body from below the neck sitting in front of themselves. They saw their own torso, but not the face and hands. **(E)** FB4 in Experiment 3: the participants saw not only the torso and legs, but also their right hand holding a paintbrush. **(F)** BP1 in Experiment 3: the subject saw the experimenter's hand from 1PP being touched by a paintbrush. **(G)** BP2 in Experiment 3: the subject saw his/her own hand from the adopted 3PP in the opposite direction (180°) holding a paintbrush and brushing the experimenter's hand. The experimenter's hand was viewed from the adopted 1PP.

The subject wore a HMD connected with a stereo camera positioned on the experimenter's head. Sitting face to face, they used their right hand to hold a paintbrush to brush each other's left hand for 2 min (Figure 1A). We call this set-up the “Basic Setting.” The brushing was either synchronous or asynchronous. In the asynchronous conditions, the subject was asked to maintain a constant speed of about 2 s per stroke, but the experimenter varied the frequency randomly from 1 to 3 s per stroke. The experimenter also randomly brushed different locations on the back of the subject's left hand, including the fingers and the wrist. Through the HMD, the subject adopted the experimenter's first person perspective (1PP) *as if* it was his/her own 1PP. We will call this *adopted 1PP*. The subject watched either the experimenter's hand from the adopted 1PP, and/or the subject's own hand from the experimenter's third person perspective (hereafter, *adopted 3PP*, 180°), or the subject's full body from the adopted 3PP (180°, with or without face).

To measure SCR, two single-use foam electrodes (Covidien, Inc., Mansfield, USA) were attached to the participant's left-hand middle finger and fourth finger, on the volar surfaces of the medial phalanges. Data were registered at a sample rate of 200 Hz, and analyzed with Biopac software AcqKnowledge v. 3.7.7. In Experiments 1 and 2, we presented a threat (kitchen knife) at the 90th second. The knife was shown in the scene and approached the stereo camera (i.e., the subject's adopted 1PP). We identified the amplitude of SCR as the difference between the maximal and minimal values of the responses within 5 s after the knife threat. Thus, what we measured was phasic SCR (Dawson et al., 2007). Those subjects who did not show any SCR amplitude were classified as non-responders, and were excluded from the analysis. Totally, we excluded 4 pieces of SCR data. After each experiment, the participant filled out a questionnaire.

Regarding statistical methods, based on many previous studies, we had strong prior expectations that the values measured in the synchronous conditions would be higher than in the asynchronous conditions, i.e., we assumed that μ_1_(synchronous) > μ_2_(asynchronous). So in Experiments 1 and 2, we used one-tailed *t*-tests to analyze both the questionnaires and SCR data. Then, to compare the sense of body-part ownership and the sense of full-body ownership, we conducted ANOVA and correlation analyses across five conditions selected from Experiments 1~3. Finally, we did a correlation analysis on the data about the sense of experiential ownership.

#### Experiment 1

In Experiment 1, the participant watched through the HMD the front side of his/her own virtual body, including not only the torso, legs, and face, but also his/her own right hand holding a paintbrush (Figure 1B). This experiment had two goals. The first was to verify whether this setting would create a variant of the full-body illusion, which may provide a new paradigm for studying full-body ownership. Second, we believe that in order to investigate the sense of experiential ownership, the experimental set-up should be arranged such that the subject may interact with another person. In Petkova and Ehrsson (2008), the subject and the experimenter squeezed each other's hands synchronously and, due to manipulation of visual perspective, some participants felt that they were shaking hands with themselves. However, Petkova and Ehrsson's research target was exclusively on the sense of body ownership. Like most studies, the sense of experiential ownership was not measured. Therefore, in Experiment 1 we used the Basic Setting to examine whether the subjective experience of “self-touching” is a solid effect, and we investigated not only the sense of body ownership but also the sense of experiential ownership. In condition 1, we performed synchronous brushing followed by a questionnaire. For the sake of later discussion, we will use “FB1” (Full-body condition 1) to indicate this condition. In condition 2 the brushing was asynchronous. Using the same set-up, in conditions 3 and 4 we measured SCR to provide objective support for conditions 1 and 2 respectively (Table 2).

#### Experiment 2

In order to exclude the possibility that the phenomena measured by Experiment 1 are merely isolated contingent effects, we applied the Basic Setting and constructed a different full-body condition. In Experiment 2, the participant watched through the HMD the front side of his/her own virtual body, including the torso and legs, but not the face. The participant also saw his/her own left hand being touched by a paintbrush held by the experimenter's hand (Figure 1C). This experiment consisted of four conditions as well, and the procedures and measurements were exactly the same as Experiment 1 (Table 2). The only difference between Experiments 1 and 2 were the HMD images described above. In Experiment 2, we will call condition 1 “FB2” (Full-body condition 2).

#### Experiment 3

We used the Basic Setting to conduct two other full-body conditions (FB3 and FB4) and two body-part conditions (BP1 and BP2). In this experiment, only the synchronous conditions were performed and measured by questionnaires. FB3: Through the HMD, the subject saw the front side of his/her own virtual body from below the neck. The subject saw his/her own torso, but not the face and hands (Figure 1D). FB4: Through the HMD, the subject saw the front side of his/her own virtual body, including not only the torso and legs, but also his/her own right hand holding a paintbrush (Figure 1E). BP1: Through the HMD, the subject saw the experimenter's hand from the adopted 1PP being touched by a paintbrush (Figure 1F). BP2: The subject saw two hands via the HMD: the subject's own hand viewed from the adopted 3PP in the opposite direction (180°) holding a paintbrush and brushing the experimenter's hand. The experimenter's hand was viewed from the adopted 1PP (Figure 1G).

## Results

### Experiment 1

**Figure 2:**
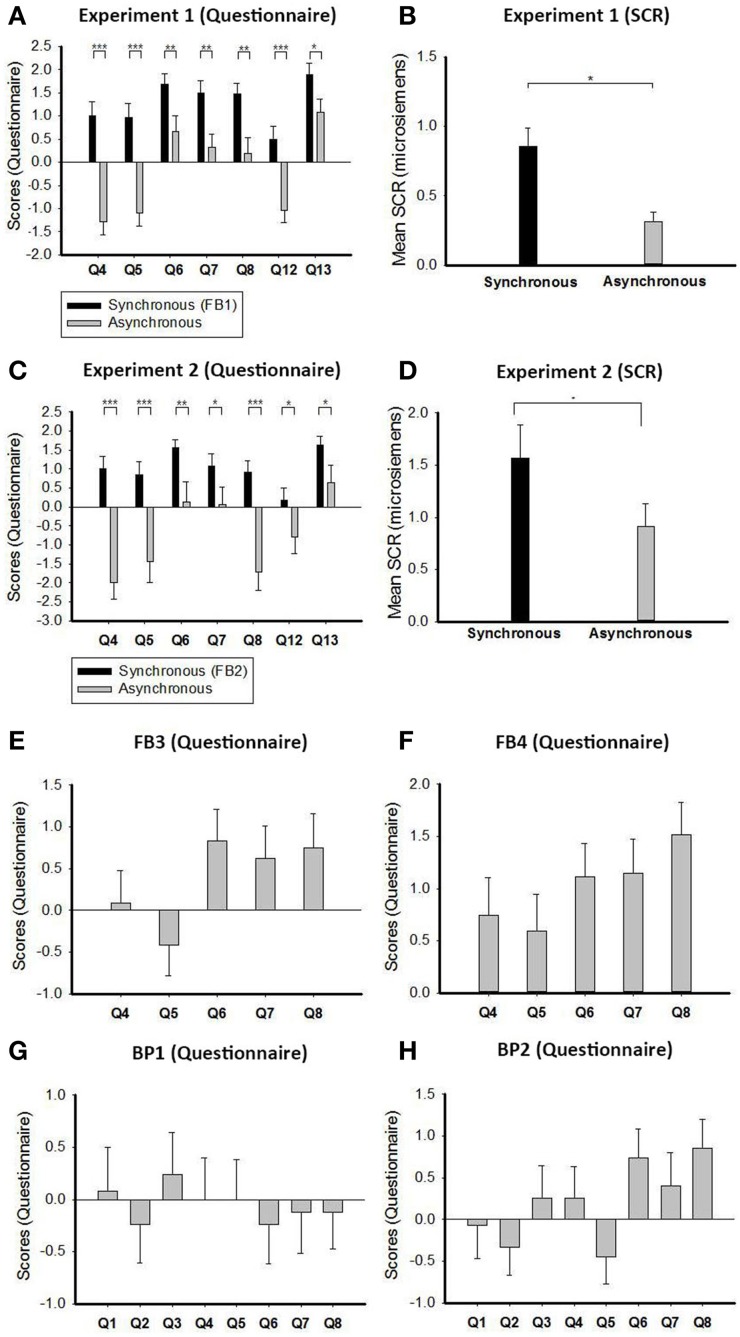
**Results**. **(A)** Questionnaire of Experiment 1. Participants indicated their responses on a scale ranging from “strongly agree” (+3) to “strongly disagree” (−3). There were significant differences between the synchronous and asynchronous conditions. **(B)** Physiological evidence of Experiment 1. SCR was measured when the subject's adopted 1PP was “threatened” with a knife. The SCR was significantly greater in the synchronous condition than in the asynchronous condition. **(C)** Questionnaire of Experiment 2. In these statements there were significant differences between the synchronous and asynchronous conditions. **(D)** Physiological evidence of Experiment 2. SCR was measured in the same way as Experiment 1. The result was significantly greater in the synchronous condition than in the asynchronous condition. **(E)** Questionnaire averages for Q4~Q8 in FB3 of Experiment 3. **(F)** Questionnaire averages for Q4~Q8 in FB4 of Experiment 3. **(G)** Questionnaire averages for Q1~Q8 in BP1 of Experiment 3. **(H)** Questionnaire averages for Q1~Q8 in BP2 of Experiment 3. For details, see the Results Section. ^*^p < 0.05, ^**^p < 0.01, and ^***^p < 0.001.

We report two key observations from Experiment 1. First, the questionnaire contained statements regarding full-body ownership (Q6), self-location (Q7), full-body agency (Q8), and the double body effect (Q12 and Q13). The average scores on these statements were significantly higher in the synchronous condition (FB1) than in the asynchronous condition (Q6: *p* = 0.0073, Cohen's *d* = 0.594; Q7: *p* = 0.0021, Cohen's *d* = 0.706; Q8: *p* = 0.0012, Cohen's *d* = 0.748; Q12: *p* = 0.0001, Cohen's *d* = 0.933; Q13: *p* = 0.0140, Cohen's *d* = 0.533, independent one-tailed *t*-test, Figure 2A). The SCR measured in conditions 3 and 4 showed the same differences as well (*p* = 0.0080, Cohen's *d* = 0.970, independent one-tailed *t*-test, Figure 2B), which provided objective support for the questionnaire data. This suggests that FB1 successfully induced a new version of the full-body illusion, where the participants felt as if the body in front of them was theirs (Q6) and that they could control it (Q8), and they felt as if they were sitting in front of their own body (Q7). These results are consistent with previous studies (Ehrsson, 2007; Lenggenhager et al., 2007). Finally, they even felt as if they had two bodies (Q12 and Q13, cf. Supplement Materials for more discussion on this effect).

Second, compared with the asynchronous condition, the synchronous full-body condition generated a “self-touching illusion”: the subject felt that “I was brushing my own hand!” This was measured by two questionnaire statements: “It felt as if I was brushing my own hand” (Q4), and “The one whom I brushed was me, not someone else” (Q5). Since Q4 and Q5 involve both hand-touching and self-identification (Blanke and Metzinger, 2009), they are associated not only with body-part but also full-body representations. Statistics showed significant differences between the synchronous and asynchronous conditions (Q4: *p* < 0.0010, Cohen's *d* = 1.301; Q5: *p* < 0.0010, Cohen's *d* = 1.168, independent one-tailed *t*-test, Figure 2A), and the SCR results provided objective evidence for this new type of full-body illusion (Figure 2B). This supports that the self-touching illusion is a distinctive version of the full-body illusion.

### Experiment 2

Using the same questionnaire in Experiment 2, we found that the average scores for full-body ownership (Q6), self-location (Q7), full-body agency (Q8), and the double body effect (Q12 and Q13) were significantly higher in the synchronous condition (FB2) than in the asynchronous condition (Q6: *p* = 0.0023, Cohen's *d* = 1.009; Q7: *p* = 0.0457, Cohen's *d* = 0.581; Q8: *p* < 0.0010, Cohen's *d* = 1.675; Q12: *p* = 0.0446, Cohen's *d* = 0.585; Q13: *p* = 0.0171, Cohen's *d* = 0.735, independent one-tailed *t*-test, Figure 2C). Also, the SCR values were significantly higher in the synchronous condition than in the asynchronous condition (*p* = 0.0473, Cohen's *d* = 0.711, independent one-tailed *t*-test, Figure 2D). This indicates that, like FB1 above, FB2 can induce a version of full-body illusion as well. These results nicely collaborate with the data collected from Experiment 1, suggesting that there are in fact many ways to induce full-body illusions.

Second, just like Experiment 1, the synchronous condition in Experiment 2, i.e., FB2, caused the subject to experience the self-touching illusion: the participants felt as if they were brushing their own hand. We observed significant differences between the synchronous and asynchronous conditions on Q4 and Q5 (Q4: *p* < 0.0010, Cohen's *d* = 1.821; Q5: *p* = 0.0003, Cohen's *d* = 1.236, independent one-tailed *t*-test, Figure 2C) and on the SCR values (Figure 2D). The data for the sense of experiential ownership will be presented later. Together with the results from Experiment 1, we confirm that the self-touching illusion is a solid effect.

### Experiment 3

Figures 2E,F show the questionnaire data of the other two full-body conditions, FB3 and FB4. Figures 2G,H present the questionnaire data of the two body-part conditions, BP1 and BP2. We will see that, by combining the data from these and other synchronous conditions, an important lesson can be drawn regarding the relationship between the sense of body-part ownership and full-body ownership.

### The sense of body ownership

One distinct feature of our study is that totally we carried out six synchronous body-part and full-body conditions, which are more than previous studies (Ehrsson, 2007; Lenggenhager et al., 2007; Petkova and Ehrsson, 2008). Another feature is that we asked self-touching questions (Q4 and Q5) and full-body questions (Q6, Q7, and Q8) both in the body-part conditions (BP1 and BP2) and in the full-body conditions (FB1~FB4). These features allow us to compare the participants' responses in many different conditions. We hypothesized that the illusory sense of full-body ownership would gradually increase from the body-part conditions to the full-body conditions. To test this hypothesis, we used ANOVA to analyze the questionnaire data on Q5~Q8 across the following series of conditions: BP1, BP2, FB3, FB4, and FB1. The order of this series was determined by the scopes that the participants saw via the HMD, which systematically increase from the body-part to the full-body conditions: BP1 (passive hand only), BP2 (both passive hand and active hand), FB3 (only torso), FB4 (torso and active hand) and FB1 (torso, active hand and face) (Figures 1B,D–G). We can see that each condition involves just one more factor than the one on its left (except for the minimum full-body condition FB3 compared with BP2) (Table 2). FB2 was not included in this analysis because the hand seen via the HMD was not on the same side compared with FB1 and FB4. We chose Q5~Q8 because they are all associated with the sense of full-body ownership, which was also why Q4 was not included. In addition to the hypothesis just mentioned, we also like to know whether significant differences will exist only between the two poles (or near the two poles) of the series, i.e., whether there will be no significant differences between any two conditions that appear next to each other in the series.

**Figure 3:**
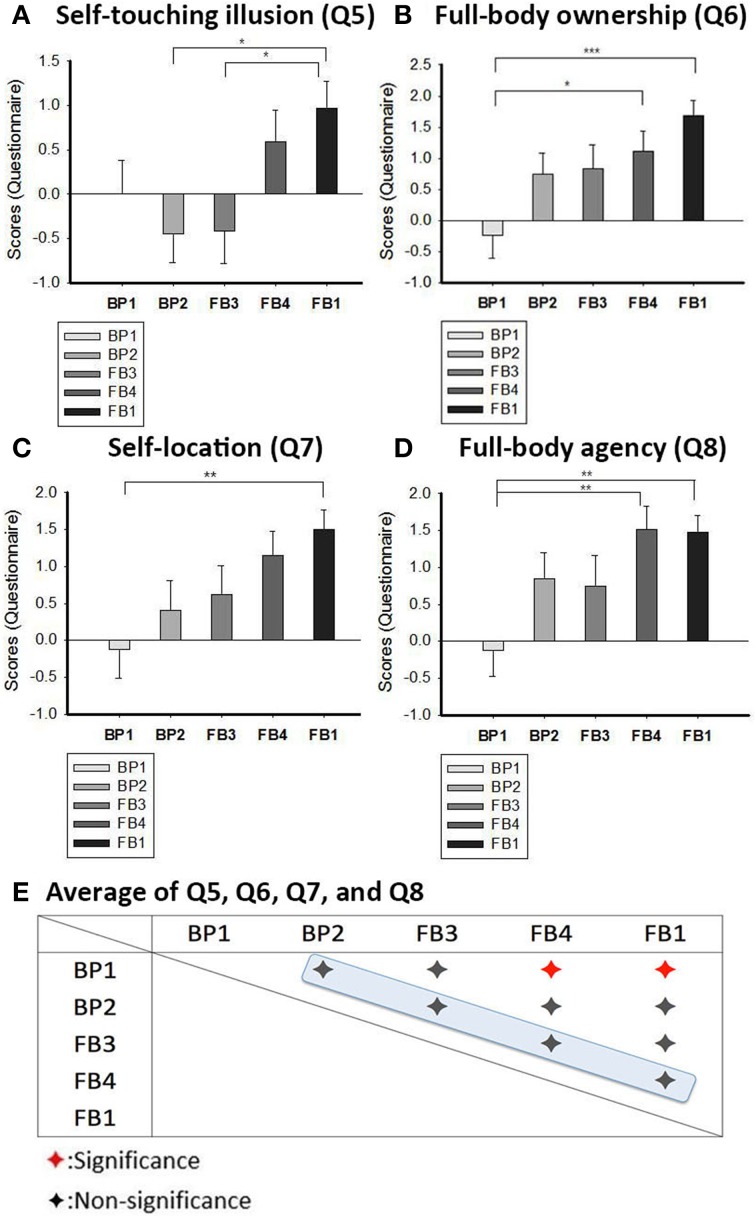
**ANOVA Results for Body Ownership**. **(A)** ANOVA result of Q5, showing means, SE, and significant relations. The star symbols represent the significant difference by *post-hoc* analysis. **(B)** ANOVA result of Q6, showing means, SE, and significant relations. The star symbols represent the significant difference by *post-hoc* analysis. **(C)** ANOVA result of Q7, showing means, SE, and significant relations. The star symbols represent the significant difference by *post-hoc* analysis. **(D)** ANOVA result of Q8, showing means, SE, and significant relations. The star symbols represent the significant difference by *post-hoc* analysis. **(E)** The whole picture of comparisons between every two conditions shown by Tukey-Kramer test result. There were no significant differences between the “neighboring conditions.”

We conducted an ANOVA analysis on Q5~Q8 to see how the answers varied across conditions. Then we did *post-hoc* analyses to know how the significances are distributed. Regarding Q5 [*p* = 0.008, *F*_(4, 136)_ = 3.625, η^2^ = 0.096, ANOVA], significant differences existed between FB1 (mean = 0.974, *SD* = 1.852) and BP2 (mean = −0.444, *SD* = 1.717) (*p* = 0.020, Tukey-Kramer test), and between FB1 and FB3 (mean = −0.417, *SD* = 1.792) (*p* = 0.032, Tukey-Kramer test) (Figure 3A). Regarding Q6 [*p* = 0.001, *F*_(4,136)_ = 5.044, η^2^ = 0.129, ANOVA], there are significant differences between FB1 (mean = 1.684, *SD* = 1.378) and BP1 (mean = −0.240, *SD* = 1.877) (*p* < 0.001, Tukey-Kramer test), and between FB4 (mean = 1.111, *SD* = 1.695) and BP1 (*p* = 0.037, Tukey-Kramer test) (Figure 3B). Regarding Q7 [*p* = 0.009, *F*_(4, 136)_ = 3.514, η^2^ = 0.094, ANOVA], significant differences existed only between FB1 (mean = 1.500, *SD* = 1.640) and BP1 (mean = −0.120, *SD* = 1.986); the *p*-value of the Tukey-Kramer test was.008 (Figure 3C). Finally, for Q8 [*p* = 0.003, *F*_(4, 136)_ = 4.219, η^2^ = 0.110, ANOVA], we observed significant differences between FB1 (mean = 1.474, *SD* = 1.428) and BP1 (mean = −0.120, *SD* = 1.787) (*p* = 0.004, Tukey-Kramer test), and between FB4 (mean = 1.519, *SD* = 1.602) and BP1 (*p* = 0.006, Tukey-Kramer test) (Figure 3D). These results support our hypothesis that significant differences are observed only between the two poles (or near the two poles) of the series, i.e., there are no significant differences between any two conditions that stand next to each other in the series (Figure 3E).

**Figure 4:**
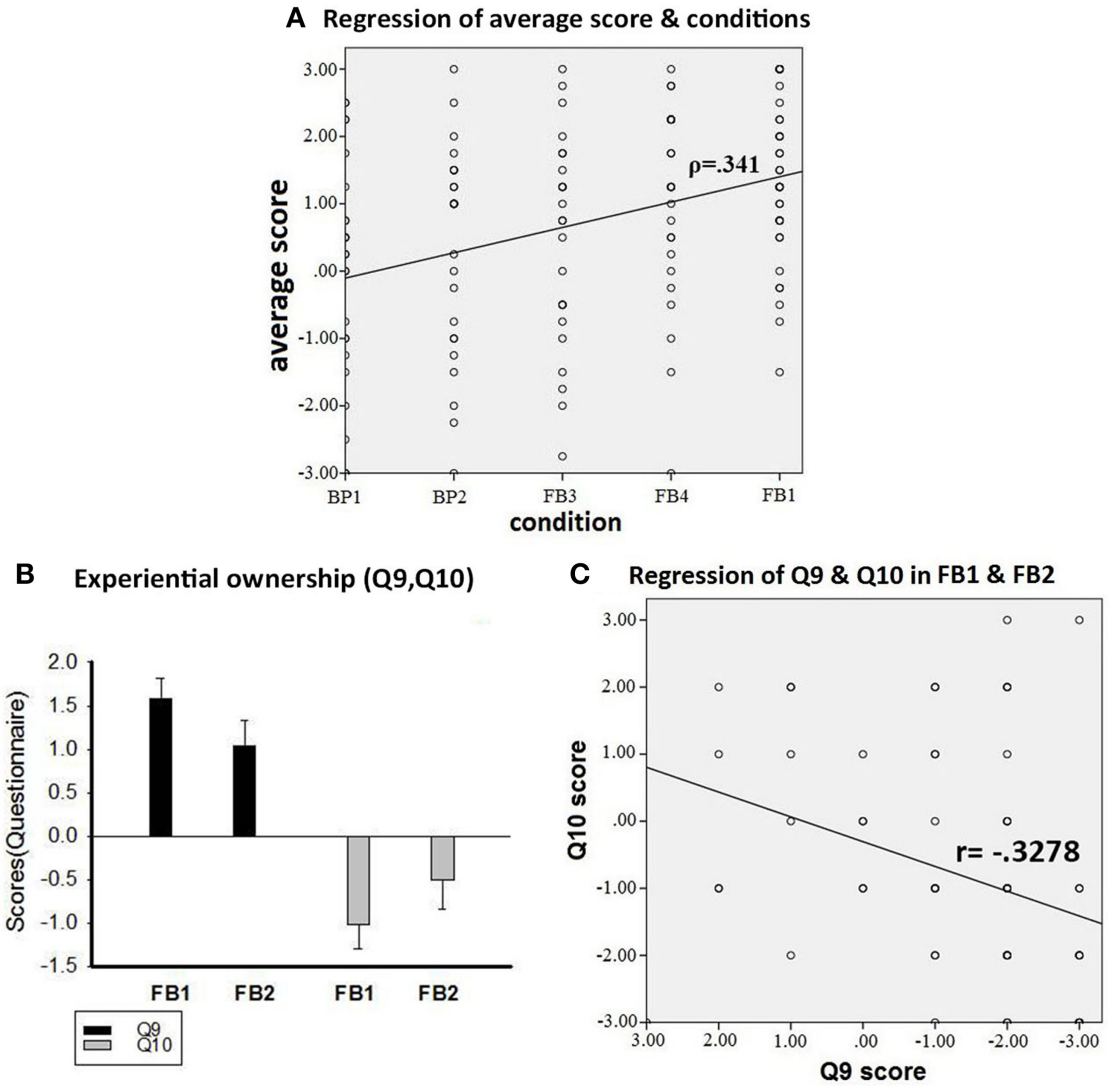
**Results of Correlation and Results of Experiential Ownership**. **(A)** Correlation between the average of Q5~Q8 and the condition variable, showing only a weakly positive correlation (ρ = 0.341). Spearman's ρ stands for the correlation coefficient. **(B)** Result of Q9 and Q10 in FB1 and FB2. Q9: “It was me who felt being brushed, not someone else.” Q10: “The person who felt being brushed was not me.” The scores were not predicted by IEM. The bars represent the mean values of the physiological scale, and the error bars indicate standard error. **(C)** indicates that the negative correlation between the scores of Q9 and Q10 in FB1 and FB2 was low (Pearson's *R* = −0.3278). For details, see the Results section.

We also did a correlation analysis on Q5~Q8 across the above five conditions, taking those conditions as a nominal variable X, and the scores of Q5~Q8 as a continuous variable Y. We found that there was a weak positive correlation between the two variables. Here are the Spearman's ρ for each of Q5~Q8: (Q5, ρ = 0.255) (Q6, ρ = 0.342) (Q7, ρ = 0.309) (Q8, ρ = 0.295). Also, the Spearman's ρ between the five conditions and the average of Q5~Q8 was low as well (ρ = 0.341, Figure 4A). All of the correlations here are significant (*p* < 0.01). Again, these results support our hypotheses that, although the illusory sense of full-body ownership gradually increases from body-part to full-body conditions, the differences between the “neighboring conditions” are not significant.

### The sense of experiential ownership

In this study, two statements were designed precisely to measure the participants' sense of experiential ownership: “It was me who felt being brushed, not someone else” (Q9), and “The one who felt being brushed was not me” (Q10). Notice that these two statements are directly opposite to each other. Also, they are not about the sense of body ownership, but about *who* felt the tactile sensations caused by brushing. We focused on FB1 and FB2, since they are supported by SCR measurements. That means the results of FB1 and FB2 revealed the participants' subjective experiences rather than just their judgments in the questionnaires. During the experiments the participants were touched by a paintbrush, so they were indeed the subjects of those tactile sensations. This fixed the *fact* of their experiential ownership. The task was to examine whether this fact was correctly represented by their *sense* of experiential ownership. We found that, in FB1 and FB2, the average scores on Q9 were 1.58 and 1.04 respectively (Figure 4B). Also, 32% of the subjects in FB1 answered (−1), (0), or (+1) on Q9, and 36% did so in FB2. The standard deviation of Q9 in FB1 was 1.50, and in FB2 it was 1.55. As an opposite statement, the average scores on Q10 in FB1 and FB2 were −1.03 and −0.50 respectively (Figure 4B). While 13.2% of participants in FB1 and FB2 disagreed with Q9 (i.e., they answered either −1, −2, or −3), 18.4% of them agreed with Q10. Figure 4C indicates that the negative correlation between these two sets of results is low (coefficient *R* = −0.3278). Later we will discuss the impact of these data on IEM and on the sense of experiential ownership.

## Discussion

### Body ownership

In our experiments, the participants not only received tactile stimulations but also held a paintbrush to touch someone else's hand. Thus, agency was clearly involved. Moreover, via the HMD, the subjects saw their own full body facing themselves (Figures 1B–E). This set-up was quite different from that in Lenggenhager et al. (2007) and Ehrsson (2007) where the participants watched their own virtual body from the back. Also, these previous studies did not involve agency; the participants only received visual-tactile stimulations either from the back (Lenggenhager et al., 2007) or in the chest (Ehrsson, 2007). Our set-up was more similar to one of the body swap experiments by Petkova and Ehrsson (2008), where the participant and the experimenter faced each other and squeezed each other's hands (cf. their Figure 6). Still, our set-up differed from theirs in that we combined a subject-experimenter interaction with the RHI. This experimental strategy—incorporating elements of body-part illusions with full-body illusions—has not been used until recently (Olivé and Berthoz, 2012; van Doorn et al., 2014). We hypothesized that the sense of body-part ownership and the sense of full-body ownership are not essentially distinct from each other. This is supported by the results of our ANOVA *post-hoc* analyses and correlation analyses reported above (Figures 3A–E, 4A). Since we address this issue only at the psychophysical level, we do not claim that our hypothesis would automatically apply at the neurophysiological level. Still, this hypothesis is useful as it can serve as a research guide or theoretical constraint for enquiries into body mereology and the relevant neural mechanisms (Petkova et al., 2011b).

Most RHI studies agree that multisensory stimulations and integration are important for explaining the illusory sense of body ownership. According to the bottom-up approach, the RHI is caused by interactions between vision, touch and proprioception (Botvinick and Cohen, 1998; Ehrsson, 2012). In contrast, the top-down approach suggests that the synchronous multisensory stimulations and integration are necessary but not sufficient for the RHI (Tsakiris and Haggard, 2005; Tsakiris, 2010, 2011). A pre-existing representation of body is required to explain various aspects of the RHI, such as the visual form congruency, anatomical congruency, postural congruency, etc., between the viewed fake hand and the felt body-part. Tsakiris proposes a model that explains the RHI in terms of three critical comparisons: “First, a pre-existing stored model of the body distinguishes between objects that may or may not be part of one's body. Second, on-line anatomical and postural representations of the body modulate the integration of multisensory information that leads to the recalibration of visual and tactile coordinate systems. Third, the resulting referral of tactile sensation will give rise to the subjective experience of body-ownership” (2010, p. 703). Not everyone agrees that there has to be a fixed body model in order to explain body ownership (Guterstam et al., 2011). We stay neutral on the debate between the bottom-up and top-down approaches, and would just like to mention that both approaches share the view that the subject's 1PP is essential for the RHI to be induced.

In contrast, the role of 1PP is more controversial in the research of full-body ownership. It has been pointed out that two rather different types of set-up have been used by researchers. As Petkova et al. (2011a, p. 2) indicate, the virtual body was either viewed by the subject from the adopted 3PP “as though looking at another individual a couple of meters in front of oneself” (Lenggenhager et al., 2007, 2009; Aspell et al., 2009), or from the adopted 1PP “as though directly looking down at one's body” (Petkova and Ehrsson, 2008; Petkova et al., 2011a; van der Hoort et al., 2011). An issue then arises concerning which set-up is more appropriate. Petkova et al. (2011a) suggested that viewing the virtual body from the subject's (adopted) visual 1PP is absolutely crucial for full-body illusions to be induced. They made the following criticism of the 3PP set-up used by Lenggenhager et al. (2007): since the virtual body was seen from 3PP and the situation is more like recognizing oneself on a surveillance monitor, what happened to the participants could be just a visual self-recognition “without necessarily experiencing a somatic illusion of ownership in the same way as in the rubber hand illusion or in the body-swap illusion” (Petkova et al., 2011a, p. 5). That is, it is possible that the participants in Lenggenhager et al. (2007) did not really experience a genuine full-body illusion.

In our full-body conditions, the virtual body was also viewed from the adopted 3PP. But we think that the criticism by Petkova et al. (2011a) can be replied to by two aspects of our experiments. First, the questionnaires in FB1 and FB2 were supported by the SCR measurements in Experiments 1 and 2, where a kitchen knife approached the subject's (adopted) visual 1PP. Although the threat was not applied to the virtual body, the SCR data collaborate well with the second aspect: we included statements about the self-touching illusion (Q4, Q5) and the double-body effect (Q12, Q13). As reported above, both the SCR and questionnaires show that significant differences exist between the synchronous and asynchronous conditions. Together, these data indicate that the participants in FB1 and FB2 experienced not only the illusory sense that they were brushing themselves, but also that they had two bodies. This goes beyond mere visual self-recognition and suggests that the relevant full-body illusions were genuinely induced.

In addition to tactile sensations, proprioception and visual 1PP, we think that there are two more factors which come into play: “visual form congruency” and “visual agency.” As we go back to consider the data revealed in Figures 3A–D, we will see that these two factors often have greater influences on the sense of body ownership than the subject's visual 1PP.

#### Visual form congruency

In our full-body conditions, the participants watched their body facing themselves. In such cases, visual form congruency refers to the scope of what the subject saw via the HMD. The scope of HMD images enlarges gradually and systematically from FB3, FB4 to FB1, which positively correlates with the strength of the relevant full-body illusions (Figures 3A–D). Although the virtual body was always presented from the adopted 3PP, this, as we have just argued above, would not necessarily hinder the relevant full-body illusions. In our body-part conditions, visual form congruency concerns whether the hand or hands that the participants saw via the HMD looked like their own. According to the first comparison in Tsakiris' model of body ownership, “the more the viewed object matches the structural appearance of the body-part's form, the stronger the experience of body-ownership will be” (2010, p. 707). We agree. Seeing one's own hand via a HMD satisfies both visual form congruency and Tsakiris' first comparison. However, as will be discussed later, our data may challenge the second comparison of Tsakiris' model, which concerns postural congruency. According to this comparison, “If there is incongruency between the posture of felt and seen hands, the seen hand will not be experienced as part of one's own body” (2010, p. 708). In our experiments, when the subject saw the experimenter's hand via a HMD, it was always presented from the subject's adopted 1PP. On the other hand, when the subject saw his/her own hand, it was always presented from the subject's adopted 3PP. We will soon consider whether postural incongruency can be remedied or outweighed by visual form congruency.

#### Visual agency

We suggest distinguishing between “body agency” and “visual agency.” Body agency refers to the subject feeling his/her own act of brushing via proprioception. It has been shown that body agency can either diminish or facilitate the RHI (Tsakiris et al., 2006; Kalckert and Ehrsson, 2012). What we would like to add is that agency can play a role in bodily self-consciousness, not only by being felt but also by being *seen*. Visual agency refers to the brushing activity that the participants *saw* through the HMD. We further suggest distinguishing between “1PP visual agency” and “3PP visual agency.” 1PP visual agency refers to the participants seeing the act of brushing by the experimenter's hand from the adopted 1PP, while 3PP visual agency refers to the participants seeing the act of brushing by their own hand from the adopted 3PP.

These two factors—visual form congruency and visual agency—can help explain the sense of body ownership involved in our experiments. Consider the series of conditions that we analyzed in the Result section: BP1, BP2, FB3, FB4, and FB1. These conditions involved the same tactile sensations, proprioception and body agency. What distinguished between them were visual form congruency and 3PP visual agency. Both the ANOVA and correlation analyses on Q6~Q8 showed that the illusory sense of full-body ownership gradually increased from the minimal body-part condition BP1 to the maximum full-body condition FB1 (Figures 3B–D). This can be nicely explained by the following comparisons: (1) compared with BP1, BP2 involves 3PP visual agency as an extra factor; (2) FB3 lacks 3PP visual agency but has a stronger visual form congruency than BP2; (3) FB4 contains not only 3PP visual agency but also a stronger visual form congruency than FB3; and (4) compared with FB4, FB1 involves an even stronger visual form congruency, i.e., seeing the subject's own face. As mentioned above, Q5 is about self-identification, and the scores across conditions seem to form a low-group (BP1, BP2, FB3) and a high-group (FB4, FB1) (Figure 3A). We suspect that this indicates that both 3PP visual agency and a strong visual form congruency are required for an illusory sense of self-identification. Notice that, even so, there are no significant differences between neighboring conditions in the series (Figure 3A). Finally, FB4 and FB1 have almost the same scores on Q8 (Figure 3D). We think this is because the factor of 3PP visual agency was the same in these two conditions. Since Q8 is about full-body agency, it is expected that 3PP visual agency would be more important than visual form congruency.

To conclude this part of the discussion, we have suggested that (1) visual form congruency can sometimes outweigh postural incongruency, which implies that the second comparison in Tsakiris' model can sometimes be violated. When there is strong visual form congruency, full-body illusions can still be induced in the face of postural incongruency; and (2) the distinction between body agency and visual agency, and the further distinction between 1PP visual agency and 3PP visual agency can help explain how body-part and full-body illusions may be hindered or facilitated.

### Experiential ownership

As mentioned above, the current mainstream view of the sense of experiential ownership is heavily influenced by Wittgenstein (1958) and Shoemaker's IEM (1968). Recall that IEM is the thesis that when I am aware of a phenomenal state through first-personal access I *cannot be wrong* about whether it is I who feels it. As mentioned in the Introduction, most of its defenders consider it as a conceptual truth based on language use. There are, therefore, very few empirical discussions on IEM and on the sense of experiential ownership (Legrand, 2007; Gallagher, 2012).

One of few exceptions was by Mizumoto and Ishikawa (2005) where the authors used a full-body illusion to argue against IEM. However, the authors described that “the subject … unanimously (all four subjects who participated in this particular experiment) reported that he ‘felt’ as if the body he was watching was his, although he in fact knew that it was not” (2005, p. 8). They also remarked that “What we have shown is the *possibility*, not the necessity, of the subject's mistakenly reacting to the attack to the other's body, which confirms our hypothesis that they felt as if they were *there* being tapped *in* the visual frame, while in fact they were not” (2005, p. 9, the authors' italics). The problem is: in our terms, they characterized their version of full-body illusion as concerning the sense of full-body ownership (and touch referral) rather than the sense of experiential ownership. As mentioned in the Introduction, IEM is about the latter, not the former. The difference between the two was nicely illustrated by two patients described by Moro et al. (2004) who denied ownership of their left hand, in which they had no sensation, and lost their left visual field. When their left hand was moved to the right so that they could see it, they became capable of detecting tactile sensations. But despite representing themselves as the ones who felt the sensations, the two patients still denied the ownership of their left hands. This shows that it is possible to have the sense of experiential ownership without the sense of body ownership.

Here, we discuss an interdisciplinary approach that defends IEM based on the phenomenological structure of experience that we call the *Pre-reflective Account* (Gallagher, 2005, 2012; Zahavi, 2005; Legrand, 2006, 2007, 2010). According to this account, the sense of self-as-subject is not achieved through introspection, judgment or attention. At the pre-reflective level, the sense of self-as-subject is a constitutive component of conscious state rather than an intentional object of consciousness. This makes the sense of self-as-subject identification-free, i.e., it does not involve identification of self as the subject, and hence enjoys IEM (Legrand, 2007; Gallagher, 2012). When I am pre-reflectively conscious of myself-as-subject, I *cannot* be wrong about whether I am the subject of experiences. We will call this view *pre-reflective immunity*. Like Shoemaker's version of IEM, pre-reflective immunity asserts a very strong modal claim. It states that violation of IEM is not possible.

Now, based on our data reported in the Results section, we argue that the sense of self-as-subject does *not* enjoy IEM, i.e., violation of IEM is possible. It is possible for misrepresentation to occur in one's pre-reflective sense of experiential ownership. If so, pre-reflective immunity does not hold. Below we show that the data of our experiments do not lend any support to Shoemaker's IEM at all. The best interpretation suggests that misrepresentation can occur in one's sense of experiential ownership. Then we respond to a possible objection to our position from the standpoint of the Pre-reflective Account.

Part of the reason why this is a knotty issue concerns how the participants understood Q9 and Q10. For the sake of argument, we will consider different possibilities: (I) Suppose the participants understood Q9 as addressing themselves. That is, from their subjective point of view: it was *me* who felt the brushing. Then, according to IEM, no participants would commit mistakes regarding their sense of experiential ownership. One would expect that most participants would answer “strongly agree” (+3) or at least “agree” (+2) on Q9. But that is not the case. 13.2% of participants in FB1 and FB2 disagreed with Q9 (i.e., they answered either −1, −2, or −3). The average scores of Q9 were much lower than this interpretation requires (Figure 4B). (II) Suppose for some reason that the participants understood Q9 as addressing someone else. That is, on their subjective experiences: it was *not me* who felt the brushing. Then, according to IEM, one would expect that most participants would answer “strongly disagree” (−3) or at least “disagree” (−2) on Q9. But this is not the case, either. This time, the average scores of Q9 are too high to fit this interpretation (Figure 4B). (III) Suppose that the participants did not all understand Q9 in the same way; some took it as addressing themselves, but others as addressing someone else. Then, assuming IEM holds, one would expect the participants to answer either +3 (or at least +2) or −3 (or at least −2). But, again, that is not the case. As reported in the Results section, many participants answered “slightly disagree” (−1), “not sure” (0), or “slightly agree” (+1). In fact, the standard deviation in each experiment is large (FB1 *SD* = 1.50, FB2 *SD* = 1.55), suggesting that the participants' responses to Q9 varied widely.

The point here is that none of the above interpretations can support IEM. Based on the data, it is more plausible that at least some participants in these experiments were uncertain about whether they were the subjects of the tactile sensations that they actually felt. This uncertainty could very well take place at the *pre-reflective level*. That is, the fact of receiving tactile sensations does not guarantee that the participants will necessarily have the *sense* that “I am the one who felt them.” There is no empirical evidence against our position here, and that our interpretation can better accommodate why the participants did not respond to Q9 in the way that conforms to IEM. The data provide empirical evidence for the possibility that one's sense of experiential ownership can misrepresent the relevant fact of experiential ownership. Hence, IEM could potentially be falsified.

The defender of pre-reflective immunity would probably reject all the above interpretations and argue that our data can be explained in a way that does not jeopardize IEM. It might be that, due to the unusual experience of the self-touching illusion, not only did different participants understand Q9 (“It was me who felt being brushed, not someone else”) differently, but also many of them were unsure about how to respond to it. The defense is that, no matter what answers the participants gave on Q9, it remains that they were the actual subjects of the tactile sensations that they felt. The variety of their answers only reveals the uncertainty of their judgments, not the uncertainty of their sense of experiential ownership or what Gallagher (2012) calls their pre-reflective 1PP. Even if some of their judgments were wrong, the mistakes were at the reflective level, not at the pre-reflective level.

Here are our responses. First, it is one thing that the participants have a pre-reflective 1PP; it is another whether they might be mistaken about that perspective. Having a pre-reflective 1PP only secures the *fact* of the participants' experiential ownership. It should not be taken for granted that this fact cannot be misrepresented by their pre-reflective *sense* of experiential ownership. Second, all the participants in our experiments were healthy subjects. There are no compelling reasons why their judgments cannot reveal their pre-reflective sense of experiential ownership. Even if they were uncertain about whom Q9 was addressing and hence were less confident about the judgments they made, this could well be an indication that at the pre-reflective level they were unsure (and hence prone to error) about who the subject of the sensations was. Finally, in addition to Q9, we also presented Q10 (“The one who felt being brushed was not me”) in the questionnaires. The direct contrast between Q10 and Q9 was so obvious that, even if the participants felt uncertain about Q9, the contrast can still be easily recognized. So, if IEM holds, one can reasonably expect that the participants' responses would manifest a strong “negative correlation” between Q9 and Q10. For example, if a subject answers +3 to Q9, then he/she would likely answer −3 (or at least −2) to Q10, etc. However, the data show no such correlation (Figure 4C).

The simple and best explanation of the data, we suggest, is that at least some of the participants were unsure or mistaken about who the subject was—*even at the pre-reflective level*. We can agree that (1) For every tactile sensation there must be a subject who experiences it; (2) Every tactile sensation is necessarily experienced from the subject's 1PP; and (3) Every tactile sensation is experienced by the one who has the 1PP of that state. However, (1)~(3) together do not imply (4): Every tactile sensation is represented from the first-person point of view *as* experienced by the one who has the 1PP of that state. The key is that (3) and (4) are not equivalent: feeling tactile sensations is one thing, but whether one experiences oneself *as* the subject of those sensations could be another. It is empirically possible that (4) was not obeyed in FB1 and FB2. This shows that violation of IEM is indeed possible.

Another possible defense of pre-reflective immunity appeals to recent studies on the second-person perspective (2PP) and social cognition (Fuchs, 2013; Froese et al., 2014). Since the experimental set-ups in FB1 and FB2 involved two people brushing each other, perhaps the brushing experience was a *social* one. A better description of the participant's experience would be: “It was *we* who felt being brushed by each other.” This can accommodate why some participants disagreed with Q9: although they agreed that “It was me who felt being brushed,” they disagreed with the latter part of Q9 “not someone else,” since there was indeed someone else, i.e., the experimenter, who felt being brushed as well. Thus, our data can be explained by the involvement of the participants' 2PP rather than by misrepresentation of their pre-reflective sense of experiential ownership.

Since we focused on the mainstream view about the sense of self-as-subject and IEM, our questionnaires did not take the second-person perspective (2PP) into account. We agree that, in future studies, it would be interesting to add 2PP statements into the questionnaires to see how the subjects respond to them. Having said this, we will make the following remarks in our defense. First, suppose *some* participants disagreed with Q9 because of the 2PP considerations, this does not mean we can be certain that *all* of those who did so were due to the same reasons. Since we argue only that IEM could potentially be falsified, this stance seems to remain intact. Second, suppose some participants' sense of experiential ownership involved a 2PP as well as a pre-reflective 1PP, and suppose that their rejection of (or uncertainty about) Q9 can be explained by the 2PP interpretation. Can we be sure that *therefore* their pre-reflective sense of experiential ownership was *necessarily* correct? Given our experiments and argument, it would require more evidence for the 2PP account to really save pre-reflective immunity from our attack. Finally, our study shows that sometimes it does make sense to ask “are you sure it is you who feel the sensations?” We think that introducing the social question “It was *we* who felt being brushed by each other” into the investigation will make it even more significant to pursue the Wittgenstein-style questions.

To conclude this part of the discussion, we have proposed a simple account three paragraphs above—(1)~(3) do not imply (4)—that challenges the mainstream view about the sense of experiential ownership. According to this account, the fact of experiential ownership can be misrepresented by the subject's pre-reflective sense of experiential ownership. Therefore, we believe that the current best evidence undercuts the empirical basis of pre-reflective immunity.

## Conclusion

We have suggested that the sense of body ownership and the sense of experiential ownership are different types of bodily self-consciousness. Regarding the former, we have proposed that (1) the self-touching illusion is a solid effect; and (2) there is no fundamental difference between the sense of body-part ownership and the sense of full-body ownership. Regarding the sense of experiential ownership, we have argued that (1) the fact of experiential ownership can be misrepresented by the subject's pre-reflective sense of experiential ownership; and (2) both Wittgenstein and Shoemaker could very well be wrong: sometimes it makes sense to ask the Wittgenstein-style questions (Q9 and Q10); it is probable that IEM as well as pre-reflective immunity fail to hold. Our study has a positive implication: not only the sense of body ownership but also the sense of experiential ownership allows and calls for interdisciplinary studies. Two important issues require further investigation. First, what is the relationship between the sense of body ownership and the sense of experiential ownership? Our current thought is that the former presupposes the latter. The idea is that when a participant experiences a body-part or a whole body as his/her own, it is relevant to consider whether the participant also represents him/herself *as* the subject of this experience of body ownership. Hence we hypothesize that the sense of experiential ownership is a constitutive component of the sense of body ownership. Further inquiries will be required to test this hypothesis. Second, what are the neural mechanisms that underlie these two types of bodily self-consciousness? Many works have been done regarding the sense of body ownership (Tsakiris, 2010; Ionta et al., 2011; Blanke, 2012; Ehrsson, 2012; Serino et al., 2013). In contrast, we currently know very little about the neural basis of the sense of experiential ownership (Christoff et al., 2011). We believe that the self-touching paradigm and the Wittgenstein-style questions that we developed can contribute to the future research on this issue.

## Author contributions

Caleb Liang designed all experiments, Si-Yan Chang, Wen-Yeo Chen, Hsu-Chia Huang, and Yen-Tung Lee conducted the experiments and analyzed the data, Caleb Liang wrote the manuscript.

### Conflict of interest statement

The authors declare that the research was conducted in the absence of any commercial or financial relationships that could be construed as a potential conflict of interest.
