# Supplementary material for: Body ownership and experiential ownership in the self-touching illusion
Source: Front Psychol. 2015 Jan 20;5:1591. doi: 10.3389/fpsyg.2014.01591 (PMC4344111; doi:10.3389/fpsyg.2014.01591)
Supplement: Supplementary file 1 [file DataSheet1.DOCX]

**Supplementary Materials**

Here we report a few secondary findings and discuss some related issues. First, we report and discuss the findings regarding body-part and full-body touch referral. Second, we discuss our observations of the double body effect. Finally, we present the supplementary data collected in Experiment 3.

1. **Touch Referral**

In the RHI studies, touch referral refers to the phenomenon whereby the subjects feel as though they were being touched not on their real hand but on the rubber hand (Botvinick and Cohen, 1998; Kammers et al., 2009) or somewhere close to the rubber hand (Folegatti et al., 2012). This has been generally considered as a strong indication of the RHI. In our study, we distinguish between body-part touch referral and full-body touch referral. The two body-part conditions **BP1** and **BP2** did not show touch referral (Suppl. Figure 1A). Since the subject was brushing the experimenter’s hand in these conditions, we think that it was body agency that hindered touch referral. We also measured touch referral in the full-body conditions (Q11: “It seemed as if the touch I felt was on the body in front of me”). We found that no full-body conditions showed touch referral (Suppl. Figure 1B). This seems to suggest that full-body touch referral is either not relevant to full-body ownership or is hindered by certain factors. Either way, we think that full-body touch referral is not necessary for inducing the illusory sense of full-body ownership. This is related to the two types of full-body experimental set-up discussed above: in the first type, the virtual body was seen from the adopted 3PP; in the second type the virtual body was seen from the adopted 1PP. We think that the reason why there was no full-body touch referral in our experiments was because the set-up that we used belongs to the first type. That is, although viewing the virtual body from the adopted 3PP did not obstruct the self-touching illusion or the illusory sense of full-body ownership, it did abolish full-body touch referral.

1. **Double body effect**

Now we report an interesting finding about the sense of full-body ownership. We explored whether during the self-touching illusion the subject might subjectively feel that they have two bodies. Two questionnaire statements were designed for this purpose: “It felt that I had two bodies” (Q12), and “It felt as if I was looking at myself from the opposite side” (Q13). It turns out that the average scores on both statements in **FB1** and **FB2** were significantly higher than the corresponding asynchronous conditions. Regarding Q12, the p-value between **FB1** and the corresponding asynchronous condition was 0.0001, and the p-value between **FB2** and its corresponding asynchronous condition was 0.0446 (independent one-tailed t-test, Suppl. Figure 1C). In the case of Q13, the p-value between **FB1** and the corresponding asynchronous condition was 0.0140, and the p-value between **FB2** and its corresponding asynchronous condition was 0.0171 (independent one-tailed t-test, Suppl. Figure 1D). With the SCR measurements described in the main text (Figure 2B and 2D), we believe that these results are significant. It suggests that the sense of full-body ownership could be as highly malleable as the sense of body-part ownership (Ehrsson, 2009; Guterstam et al., 2011). It is possible for healthy participants to have subjective experiences of owning two bodies. This result also indicates that the self-touching experiments in this study provide a useful approach to studying the sense of full-body ownership.

In a recent study by Guterstam and Ehrsson (2012), the participants watched their own back via a HMD from 2 meters behind, and synchronous visuo-tactile stimulations induced an illusory sense of self-location. But when the real body was threatened, the SCR was significantly lower in the synchronous than in the asynchronous condition. Guterstam and Ehrsson took these results to mean that, while the participants “experienced ownership of an illusory body at the locations of the cameras,” they at the same time “disowned their veridical body” (2012, 1040). The authors concluded that “it might not be possible for a healthy brain to perceive the self to be located at two different places at the same time and owning two different bodies at these locations” (2012, 1041). However, as reported above, our experiments **FB1** and **FB2** suggested a “double body effect”: the participants illusorily experienced that they had two bodies. A live issue therefore arises concerning whether the double body effect is genuine or even possible.

Notice that both Guterstam and Ehrsson’s set-up and ours belong to the first type mentioned above. But, while we measured SCR only by threatening the subject’s adopted 1PP, they did so only by threatening the subject’s adopted 3PP. In our study, the participants saw their own body facing themselves, and the body was seen as quite close to their adopted 1PP (Figure 1A) compared to the 2-meter distance in Guterstam and Ehrsson’s case. These differences should be examined in future studies. Although we do not claim to solve the issue here, we will make the following remarks in our defense. First, the double body effect that we observed was not an isolated phenomenon. It was observed in two different conditions, **FB1** and **FB2**. The results were supported by the SCR data, and were consistent with our measurements of the self-touching illusion and the illusory sense of full-body ownership reported in the main text. Second, our observation is also consistent with a recent study by Heydrich et al. (2013) in which they used two different methods—a HMD-camera set-up and virtual reality techniques—to induce the experience of owning two bodies. Finally, in two previous studies by Ehrsson’s group, an illusory sense of owning a third arm was generated by variant types of the RHI (Ehrsson, 2009; Guterstam et al., 2011). Given our hypothesis that there is no fundamental difference between the sense of body-part ownership and the sense of full-body ownership, these two studies seem to support that the double body effect is both theoretically and empirically possible. Therefore, based on these considerations, we think that the double body effect measured in our experiments is a genuine effect, and that the sense of full-body ownership is more malleable than Guterstam and Ehrsson (2012) have suggested.

1. **Supplementary data in Experiment 3**

Suppl. Figures 1E and 1F present the questionnaire data collected in Experiment 3 that are not in the main text.

**References**

Botvinick, M., and Cohen, J. (1998). Rubber hands 'feel' touch that eyes see. *Nature*, *391*(6669), 756. doi: 10.1038/35784.

Ehrsson, H. H. (2009). How many arms make a pair? Perceptual illusion of having an additional limb. *Perception*, *38*(2), 310-312. doi: 10.1068/p6304

Folegatti, A., Farnè, A., Salemme, R., de Vignemont, F. (2012). The rubber hand

illusion: Two’s a company, but three’s a crowd. *Consciousness and cognition*, 21(2), 779-812. doi: 10.1016/j.concog.2012.2.008

Guterstam, A., Petkova, V. I., and Ehrsson, H. H. (2011). The illusion of owning a third arm. *PLoS One*, *6*(2), e17208. doi: 10.1371/journal.pone.0017208

Guterstam, A., and Ehrsson, H. H. (2012). Disowning one’s seen real body during an out-of-body illusion. *Consciousness and cognition*, *21*(2), 1037-1042. doi: 10.1016/j.concog.2012.01.018

Heydrich, L., Dodds, T. J., Aspell, J. E., Herbelin, B., Bülthoff, H. H., Mohler, B. J., and Blanke, O. (2013). Visual capture and the experience of having two bodies–Evidence from two different virtual reality techniques. *Frontiers in psychology*, *4*. doi: 10.3389/fpsyg.2013.00946

Kammers, M. P. M., de Vignemont, F., Verhagen, L., and Dijkerman, H. C. (2009). The rubber hand illusion in action. *Neuropsychologia*, *47*(1), 204-211. doi: 10.1016/j.neuropsychologia.2008.07.028


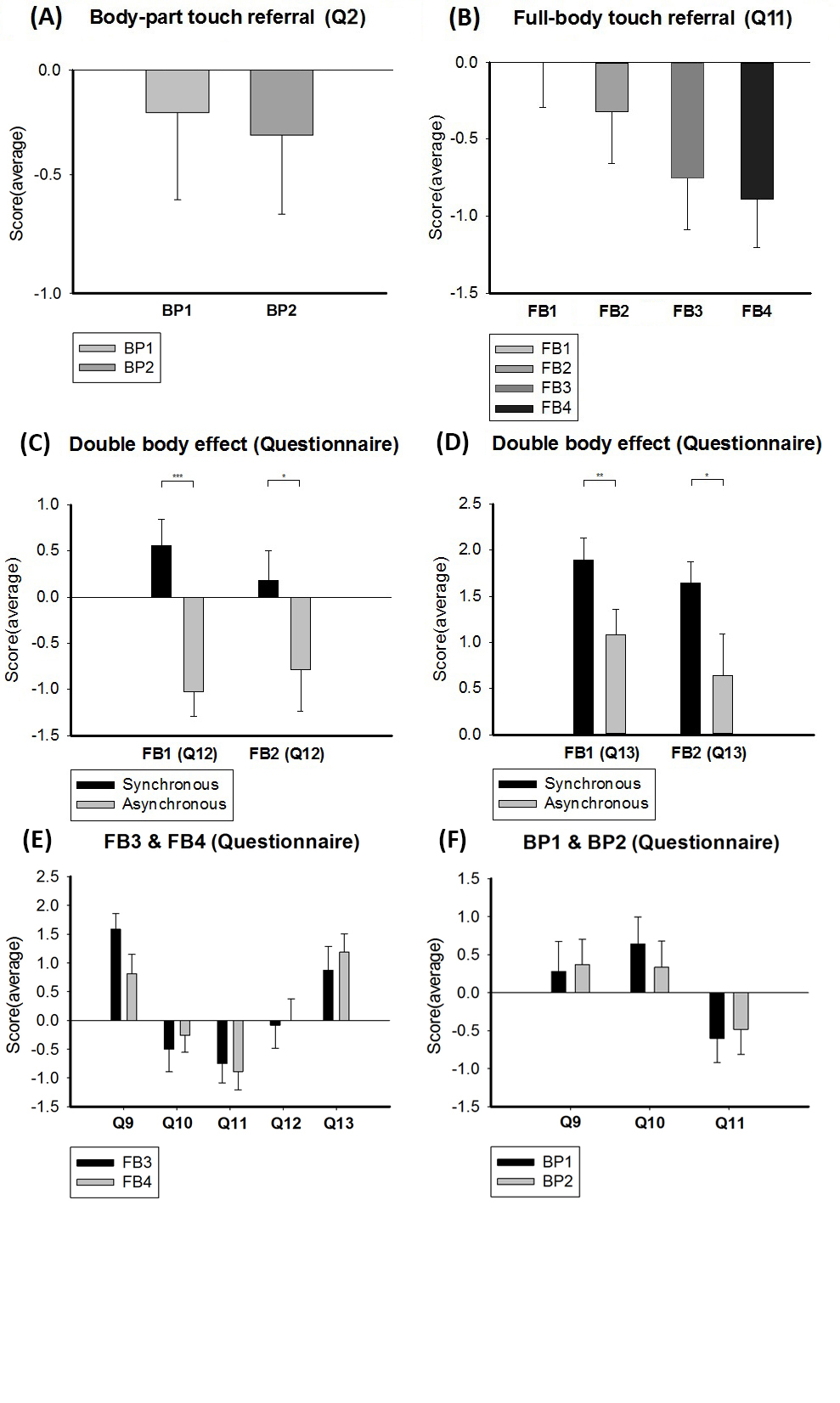


**Suppl. Figure 1. Questionnaire results. (A)** The result of the body-part touch referral statement. Q2: “It seemed as if the touch I felt was on the hand brushed by the paintbrush in the screen.” Touch referral was hindered by body agency involved in **BP1** and **BP2**. **(B)** The result of the full-body touch referral statement. Q11: “It seemed as if the touch I felt was on the body in front of me.” No full-body conditions exhibited touch referral, suggesting that full-body touch referral is not necessary for the illusory sense of full-body ownership. **(C)** The result of a double-body statement in two full-body conditions. Q12: “It felt that I had two bodies.” The result showed significant differences between the synchronous (**FB1** & **FB2)** and asynchronous conditions (p<.01). **(D)** The result of another double-body statement. Q13: “It felt as if I was looking at myself from the opposite side.” There were significant differences between the synchronous (**FB1** & **FB2)** and asynchronous conditions (p<.05) as well. **(E)** The statistical results of the average score of Q9~Q13 in **FB3** & **FB4**. **(F)** The statistical results of the average score of Q9~Q11 in **BP1** & **BP2**.
